# Identifying daily changes in circRNAs and circRNA-associated-ceRNA networks in the rat pineal gland

**DOI:** 10.7150/ijms.51743

**Published:** 2021-01-14

**Authors:** Yi Zheng, Hao Jiang, Hao-Qi Wang, Hai-Xiang Guo, Dong-Xu Han, Yi-Jie Huang, Yan Gao, Bao Yuan, Jia-Bao Zhang

**Affiliations:** Department of Laboratory Animals, Jilin Provincial Key Laboratory of Animal Model, Jilin University, Changchun 130062, Jilin, P.R. China.

**Keywords:** Pineal gland, RNA sequencing, microRNAs, circular RNAs

## Abstract

Circular RNAs (circRNAs) are a new class of covalently closed circular RNA molecules that are involved in many biological processes. However, information about circRNAs in the pineal gland, particularly that of rats, is limited. To establish resources for the study of the rat pineal gland, we performed transcriptome analysis of the pineal glands during the day and night.

In this study, 1413 circRNAs and 1989 miRNAs were identified in the pineal gland of rats during the night and day using the Illumina platform. Forty differentially expressed circRNAs and 93 differentially expressed miRNAs were obtained, among which 20 circRNAs and 37 miRNAs were significantly upregulated during the day and 20 circRNAs and 56 miRNAs were significantly upregulated during the night.

As circRNAs have been reported to work as miRNA sponges, we predicted 15940 interactions among 40 circRNAs, 93 miRNAs and 400 mRNAs with differential diurnal expression using miRanda and TargetScan to build a ceRNA regulatory network in the rat pineal gland.

The diurnal expression profile of circRNAs in the rat pineal gland may provide additional information about the role of circRNAs in regulating changes in melatonin circadian rhythms. The analyzed data reported in this study will be an important resource for future studies to elucidate the altered physiology of circRNAs in diurnal rhythms.

## Introduction

The pineal gland is one of the central organs that regulates the circadian system. It converts external light signals into endogenous signals by secreting melatonin, the hormone of the night. In mammals, the nerve endings of sympathetic axons in the superior cervical ganglia (SCG) release norepinephrine (NE) and control melatonin synthesis 1. NE stimulates the alpha and beta adrenergic receptor. The alpha adrenergic receptor stimulation increases intracellular Ca^2+^ concentration and the beta adrenergic receptor activates the adenylate cyclase (AC)/cyclic adenosine monophosphate (cAMP)/protein kinase A (PKA) pathway, which converts the basic leucine zipper (bZIP) TF cAMP-responsive element binding protein (CREB) into its phosphorylated form (pCREB) 2, 3. At night, the cAMP/PKA/pCREB pathway 4 and other NE-stimulated signaling mechanisms 5 play a role in increasing the expression and activity of aralkylamine N-acetyltransferase (AANAT) and acetylserotonin O-methyltransferase (ASMT) 6-8, which are the last two enzymes in melatonin synthesis. Then cAMP-dependent phosphorylation triggers the binding of AANAT to 14-3-3 proteins (30 and 33 kDa), a family of highly conserved isoforms that exist as dimers. The pAANAT/14-3-3 complex shields AANAT from metabolic processes that lead to proteolysis. And the AANAT enzyme activity are more than 100-fold increased. Fianlly this mechanism significantly increase melatonin secretion. Whereas the regulation of melatonin secretion in the pineal glands of fish, amphibians, reptiles, and birds is different from that in mammals 9, the rat is one of the preferred experimental species to study the role of the pineal gland in melatonin secretion and some melatonin-deficient diseases in humans, such as adolescent idiopathic scoliosis 10, mammary tumorigenesis 11, Alzheimer's disease 12 and hypoxic-ischemic brain damage 13.

In addition to mRNAs, relevant experiments have shown that microRNAs (miRNAs) are also involved in melatonin secretion from the pineal gland. In the rat pineal gland, miR-483 was the first miRNA found to inhibit melatonin secretion 14. And the four most abundant miRNAs are miR-182, miR-183, miR-127 and miR-96 and account for nearly half of the total miRNA population in the pineal gland. Then, miR-182 and miR-325-3p were found to regulate rat pineal function with HIBD 13, 15. LncRNAs were also found to act as miRNA sponges to activate the CLOCK gene in the rat pineal gland 16. However, studies on circular RNAs (circRNAs) in the rat pineal gland are still very rare.

CircRNAs have recently been identified as a new type of ncRNA 17. CircRNAs are closed RNA molecules that are mainly produced from premRNA through variable shear processing in the eukaryotic transcriptome 18. The circRNA ring structure makes it less sensitive to RNA exonuclease (RNase R) than linear RNA 19. CircRNAs are conserved in different species and are abundantly expressed in the brain 20. As shown in previous studies, circRNAs play important regulatory roles at the transcriptional and posttranscriptional levels 21. CircRNAs also serve as competitive endogenous RNAs (ceRNAs) by binding intracellular miRNAs and blocking the inhibitory effects of miRNAs on target genes 22; for example, circRNA-ZFR functions as a miR-101-3p sponge to increase *Cullin 4B* expression 23. Relevant studies have also found that very few circRNAs are directly translated 24, 25; for example, one such circRNA, circPPP1R12A, encodes a novel protein that promotes colon cancer. Although circRNAs have important biological functions, the roles of circRNAs in the pineal gland remain to be explored. Therefore, the expression and functions of circRNAs in the pineal gland must be identified.

This study explored the diurnal expression of circRNAs, miRNAs and mRNAs in the rat pineal gland using RNA-seq. The sequencing results were used to analyze, predict and construct possible ceRNA interaction networks. These results may identify new functions and roles of circRNAs in the pineal gland and provide ideas for further research into the mechanisms of ncRNAs in this tissue.

## Materials and methods

### Ethics statement

All animal procedures were performed according to the guidelines of the Guide for the Care and Use of Laboratory Animals of Jilin University, and the experimental protocols were approved by the Institutional Animal Care and Use Committee of Jilin University, Changchun, China. The permit number was SY201901011.

### Animals

Sprague-Dawley (SD) rats were obtained from Liaoning Changsheng Biotechnology Co., Ltd. The pineal gland and other tissues were extracted from eight-week-old male SD rats. The rats were acclimated at room temperature (21±2 °C) on a 12:12 h light/dark cycle for at least 1 week with free access to food and water. All rats used in this study were euthanized with a CO_2_ anesthesia machine. CO_2_ levels were increased to 99.9% at a rate of 30% per minute and maintained at this level for 10 minutes to ensure that all rats were euthanized. Some rats were anesthetized and sacrificed in bright sunlight at Zeitgeber time (ZT) 7 and were considered the light group. Three of these rats were named C01, C02 and C03 (or S01, S02, and S03). Other rats were anesthetized and sacrificed under dark conditions (under red light) at ZT 19 and were considered the dark group. Three of these rats were named C04, C05 and C06 (or S04, S05, and S06). The rats named C01~06 were used for RNA-seq, and the other rats were used for other experiments.

### RNA isolation

Total RNA was isolated using TRIzol reagent (Invitrogen). RNA degradation and contamination, particularly DNA contamination, were monitored on 1.5% agarose gels. RNA concentrations and purity were measured using a NanoDrop 2000 spectrophotometer (Thermo Fisher Scientific, Wilmington, DE). RNA integrity was assessed using the RNA Nano 6000 Assay Kit and the Agilent Bioanalyzer 2100 System (Agilent Technologies, CA, USA).

### Library preparation for sequencing

For circRNAs and mRNA, the Ribo-Zero rRNA Removal Kit (Epicenter, Madison, WI, USA) was used to remove rRNA. Sequencing libraries were generated using the NEBNext^®^ Ultra^TM^ Directional RNA Library Prep Kit for Illumina^®^ (NEB, USA) according to the manufacturer's recommendations, and index codes were added to attribute sequences to each sample. After adding fragmentation buffer to break the rRNA-depleted RNA at random, first-strand cDNAs were synthesized using random hexamer primers and reverse transcriptase. Then, second-strand cDNA synthesis was subsequently performed using DNA polymerase I and RNase H. The remaining overhangs were converted into blunt ends. After adenylation of the 3' ends of DNA fragments, the NEBNext adaptor with a hairpin loop structure was ligated to prepare for hybridization. AMPure XP Beads (Beckman Coulter, Beverly, USA) were used to preferentially select insert fragments with a length of 150-200 bp. Then, PCR was performed with Phusion High-Fidelity DNA polymerase, Universal PCR primers and Index(X) Primer.

For miRNAs, a total amount of 2.5 ng of RNA per sample was used as input material for RNA sample preparation. Sequencing libraries were generated using NEBNext® Small RNA Library Prep Set for Illumina (NEB, USA) according to the manufacturer's recommendations, and index codes were added to attribute sequences to each sample. Briefly, first, the 3' SR adaptor was ligated. The SR RT primer hybridizes to the excess of the 3' SR adaptor and transforms the single-stranded DNA adaptor into a double-stranded DNA molecule to prevent the formation of adaptor dimers. Second, the 5' SR adaptor was ligated. Then, reverse transcription was performed to synthesize the first chain. Finally, PCR amplification and size selection were conducted. A PAGE gel was used to electrophoretically screen fragments, and gel extraction was performed on the fragments to obtain small RNA libraries.

The PCR products were subsequently purified (AMPure XP system), and the library quality was assessed using the Agilent Bioanalyzer 2100 system.

### Clustering and sequencing

The clustering of the index-coded samples was performed on the cBot Cluster Generation System using the TruSeq PE Cluster Kit v3-cBot-HS (for circRNA and mRNA) and v4-cBot-HS (for miRNA) (Illumina) according to the manufacturer's instructions. After cluster generation, the library preparations were sequenced on an Illumina HiSeq X Ten platform, and 2*150-bp paired-end reads (for circRNA and mRNA) and 50-nt single-end reads (for miRNA) were generated. The construction of the libraries and the RNA-Seq were performed by the Biomarker Biotechnology Corporation (Beijing, China). The sequencing data were uploaded in GSE146193.

### Quality control for libraries

Raw reads in the fastq format were initially processed using in-house Perl scripts. In this step, clean reads were obtained by removing reads containing adapters and poly-N sequences and low-quality reads from the raw data.

For miRNAs, reads were trimmed and cleaned by removing sequences smaller than 15 nt or longer than 35 nt. At the same time, the Q-score and GC content of the clean data were calculated 26. Q-score = -10*log_10_ P (in the formula, P is the probability of error in base recognition). All downstream analyses were based on high-quality clean reads. At the same time, Pearson's correlation coefficient was calculated to evaluate correlations between biological replicates of the sequencing samples 27.

After clean reads were obtained, they were aligned with the rat genome to obtain the location in the rat genome (Rnor_6.0) and the unique sequence characteristics of the sequencing sample. The reads that mapped to the specific site in the rat genome are called mapped reads, and the corresponding data are called mapped data.

### Identification of circRNAs, mRNAs and miRNAs

We used CIRI (CircRNA Identifier) tools and find_circ software to identify circRNAs 28, 29. CIRI software uses the reads to compare with the reference gene sequence, generates SAM files, analyzes the CIGAR values in SAM files, and scans PCC signals (paired chiastic clipping signals) from SAM files. The CIGAR value in the junction read is characterized by xS/HyM or xMyS/H, where X and y represent the number of bases, M is on mapping, S is soft clipping, and H is hard clipping. For double-end reads, the CIRI algorithm considers a pair of reads, one of which can be mapped to circRNA, and the other one also needs to be mapped to circRNA's sequence interval. For single exons forming rings, or the circular structure formed by “long exon 1-short exon - long exon 2”, CIGAR values should be xS/HyMzS/H and (x+y)S/HzM or xM(y+z)S/H. CIRI software can separate these two cases very well. For splicing signals GT/AG, CIRI will also consider other weak splicing information, such as AT/AC, and the algorithm will extract exon boundary positions from the GTF/GFF file and filter false positives with known boundaries. Since the ring-forming splicing sites of the circRNA reads could not be directly aligned to the genome, the find_circ software first took 20 bp as anchor points at both ends of reads that could not be aligned with the genome and then took anchor points as independent reads to the genome for alignment and searched for the unique matching sites. If the alignment position of two anchor points is reversed in the linear direction, the reads of anchor points are extended until the junction position of circRNA is found. At this time, the sequences of both sides are GT/AG splicing signals, and the circRNA reads are judged to be circRNAs.

For mRNA, the transcriptome was assembled using StringTie based on the reads mapped to the reference genome. The assembled transcripts were annotated using the gffcompare program. Three computational approaches, including CPC/CNCI/Pfam/CPAT, were combined to sort nonprotein coding RNA candidates from putative protein-coding RNAs in the unknown transcripts. Putative protein-coding RNAs were filtered out using a minimum length and exon number threshold. Transcripts with lengths greater than 200 nt and more than two exons were selected as lncRNA candidates and further screened using CPC/CNCI/Pfam/CPAT, which has the power to distinguish protein-coding genes from noncoding genes.

The intersection of the results obtained using the two methods was the final prediction. In terms of the identification of known miRNAs, we compared the read sequences with the reference genome using the mature miRNA column in miRBase 30. Read sequences that were identical to known miRNAs were considered known miRNAs. For sequences that have not been identified as known miRNAs, miRDeep2 software was used to predict new miRNAs 31.

### Analysis of differentially expressed RNAs

For circRNA, miRNA and mRNA, the expression levels were determined from the read counts and were normalized using the transcripts per million (TPM) algorithm 32. The differential expression analysis was performed for the two groups (C01, C02, C03 and C04, C05, C06) using the DESeq R package. CircRNAs with an adjusted *P* value <0.05 and an absolute value of a log2 (fold change) >2 and miRNAs with an adjusted *FDR* <0.01 and an absolute value of a log2 (fold change) >0.5 identified using DESeq were considered differentially expressed.

### Prediction of miRNA target genes

The miRanda (animal), RNAhybrid and TargetScan databases were used to predict the miRNAs targeting mRNAs and circRNAs. The input files were miRNA, mRNA and circRNA base sequence files that we obtained from the RNA-seq. Then, the circRNAs, mRNAs and miRNAs with different expression were selected to construct the ceRNA network.

### Functional annotation of circRNA host genes and miRNA target genes

Some circRNA sequences can be aligned to a gene sequence, which is defined as the circRNA host gene. The functions of circRNA host genes and miRNA target genes were annotated using the NR database 33 (a nonredundant protein sequence database), the Swiss-Prot database 34 (a manually annotated, nonredundant protein sequence database), the Gene Ontology (GO) database 35, the Clusters of Orthologous Groups of proteins (COG) database 36, the euKaryotic Orthologous Groups of proteins (KOG) database 37, and the Kyoto Encyclopedia of Genes and Genomes (KEGG) database 38.

### Reverse transcription and qPCR

First-strand cDNAs were generated using a FastKing RT Kit (with gDNase) (Tiangen) with random primers according to the manufacturer's protocol. Primers for the differentially expressed circRNAs were obtained from RiboBio Biotechnology Co., Ltd (Support Table S13). qPCR was performed using a Mastercycler ep Realplex2 system (Eppendorf, Germany). The products were quantified using Super Real PreMix Plus (SYBR Green, Tiangen, China) according to the manufacturer's instructions. The relative expression levels of circRNAs were normalized to GAPDH, and the dark group was considered the control group.

### Statistical analysis

All data are presented as the means ± standard deviations of three independent experiments. Significant differences between multiple groups were determined using one-way ANOVA with SPSS 19.0 for Windows. *P* < 0.05 was considered a statistically significant difference.

## Results

### Sequencing quality control

The original sequences obtained from sequencing contain joint sequences or low-quality sequences. Quality control of the original data was conducted to obtain high-quality sequences (i.e., clean reads) and to ensure the accuracy of the subsequent analysis. After sequencing quality control, a total of 127.99 Gb clean data were obtained for circRNA samples. Per sample has an average of 71.3 M reads, and the Q-score (30) percentage of each sample was not less than 95.05% (Support Table S1). For the miRNAs, the number of clean reads in each miRNA sample was greater than 17.75 M after quality control, and the Q-score (30) percentage of each sample was not less than 98.32% (Support Table S2).

Sequence alignment and subsequent analysis were performed using the rat genome as a reference. Clean reads were sequentially aligned with the rat genome to obtain the location in the rat genome or gene, as well as the characteristic information about the sequence that was unique to the sample.

According to the comparison of circRNA sequencing results, the ratio of reads to the rat genome reached 99.99% (Support Table S3). Clean reads obtained from miRNA sequencing were sequentially compared with the Silva database, GtRNAdb database, Rfam database and Repbase database using Bowtie software. The reads were categorized and annotated as scRNA, rRNA, snRNA, snoRNA, tRNA and unannotated reads containing miRNA sequences. The results showed that an average of 67% of the unannotated reads were identified as miRNAs (Support Table S4). Data utilization was normal, and the selected reference genome was sufficient for subsequent analysis.

**Figure 1:**
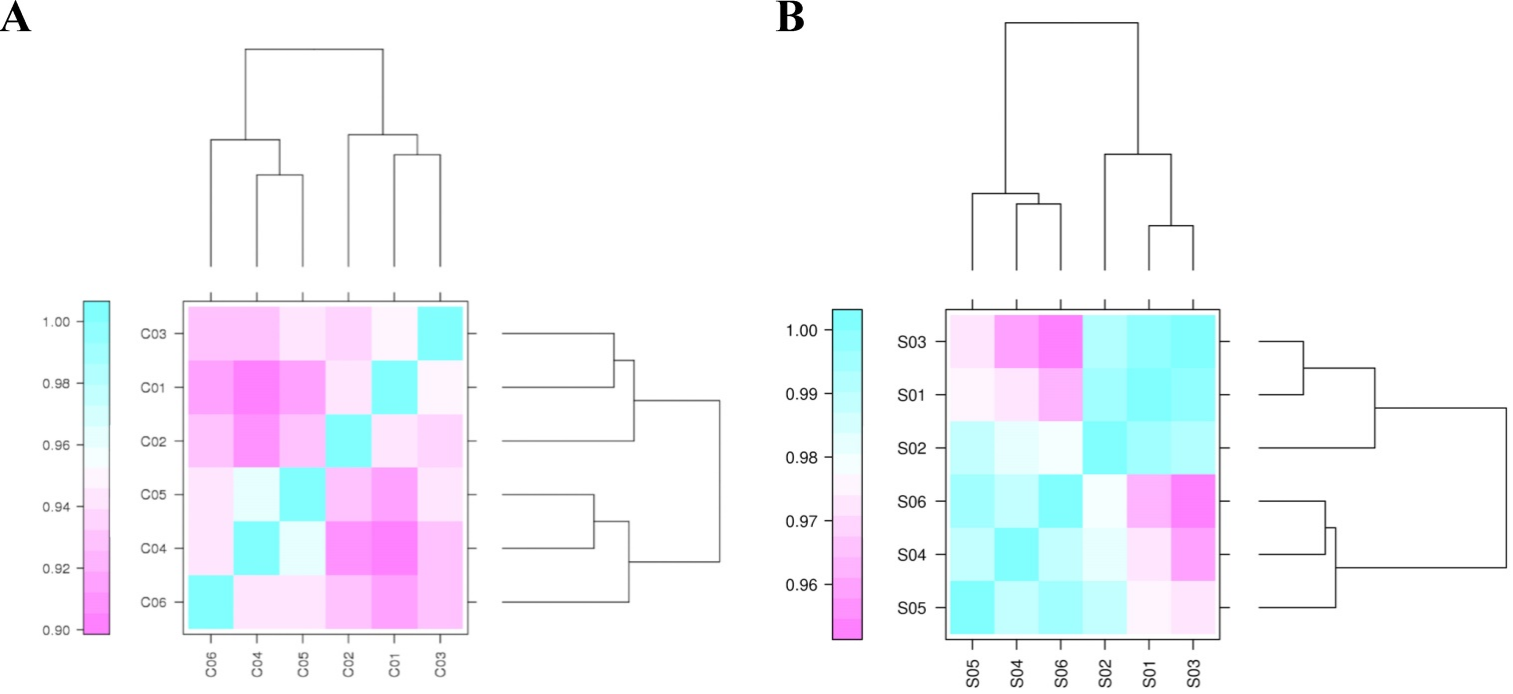
** Correlation coefficients for circRNA and miRNA samples.** “C” represents the sample for circRNA-seq. C01, C02, and C03 indicate the samples harvested under light conditions. C04, C05, and C06 represent the samples collected under dark conditions. “S” indicates the sample for miRNA-seq. S01, S02, and S03 are the samples collected under light conditions. S04, S05, and S06 represent the samples collected under dark conditions.

An assessment of the correlation of biological replicates is important for analyzing transcriptome sequencing data. It tests the repeatability of biological experiments, evaluates the reliability of differentially expressed genes, and assists with screening abnormal samples. Pearson's correlation coefficient (r) was calculated to evaluate the correlations of biological replicates. The closer r2 is to 1, the stronger the correlation between the two repeated samples. The r2 statistics of any pair of circRNA and miRNA samples are shown in Figure 1, Support Table S5 and S6. Most circRNAs and miRNAs in the two groups showed similar expression trends and a high replicate correlation (all r2 values were above 0.90).

### Deep sequencing analysis of circRNAs and miRNAs in rats

**Figure 2:**
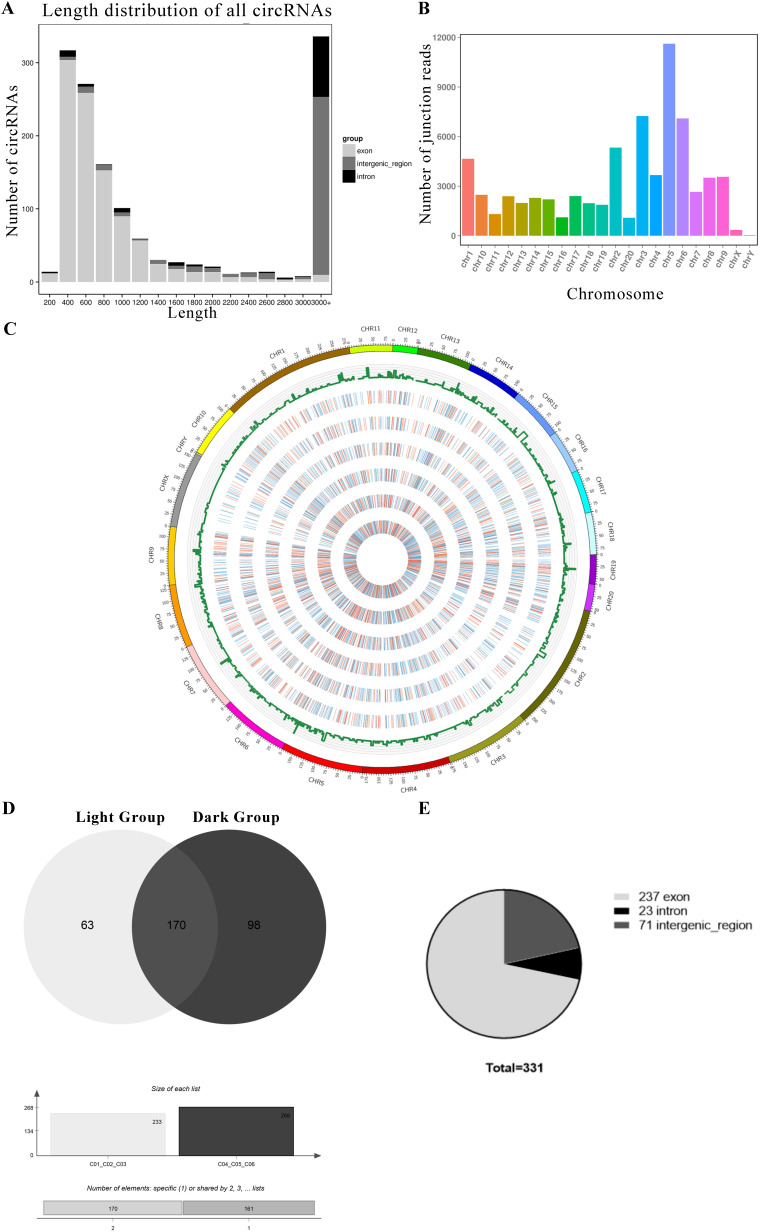
**The circRNAs exhibiting diurnal expression patterns in the rat pineal gland.** (A) Length distribution of all circRNAs. (B) Distributions of all circRNAs on chromosomes. (C) Statistical analysis of the circRNA distribution in the genome and a circos plot of the expression levels in each sample. The histogram shows the number of circRNAs at each position, and the color represents the expression distribution of each sample: 0-1: blue, 1-10: green, 10-100: orange, and 100+: red. The heat map from the outside to the inside indicates C01, C02, C03, etc. (D) Diagram of circRNAs that are expressed in all three replicates of a group (light or dark). (E) Numbers of the three types of circRNAs coexpressed in the light group and dark group.

We performed RNA-seq to detect circRNA and miRNA expression in rat pineal glands between day and night. We found that a total of 1413 circRNAs were expressed in the pineal gland (Support Table S7). The circRNAs were named according to the positions on the genome that the software recognizes on both ends of their base sequences. The lengths of the circRNAs were distributed from 200-3000+ nt. Most circRNAs were exonic circRNAs, but circRNAs with lengths greater than 3000 nt were mainly derived from intergenic regions (Figure 2A). Although the circRNAs were distributed among all 22 chromosomes, they were mainly distributed on chromosomes 3, 5 and 6 (Figure 2B). Then, we mapped the measured circRNAs to the genome and drew a circos diagram (Figure 2C).

However, not all the circRNAs were both expressed in the light group and dark group, and we selected the circRNAs expressed in all three replicates of the light group or the dark group to be used in the next analysis. Sixty-three circRNAs were mainly expressed under light conditions. They were expressed in all light samples and not expressed in at least one dark sample. Ninety-eight circRNAs were mainly expressed under dark conditions. In addition, 170 circRNAs were coexpressed in all samples (Figure 2D). The selected circRNA sources were statistically analyzed, and 72% of circRNAs were derived from exons, 7% were derived from introns, and 21% were derived from intergenic regions (Figure 2E). The relevant mRNA expression information is provided in Support File 1.

**Figure 3:**
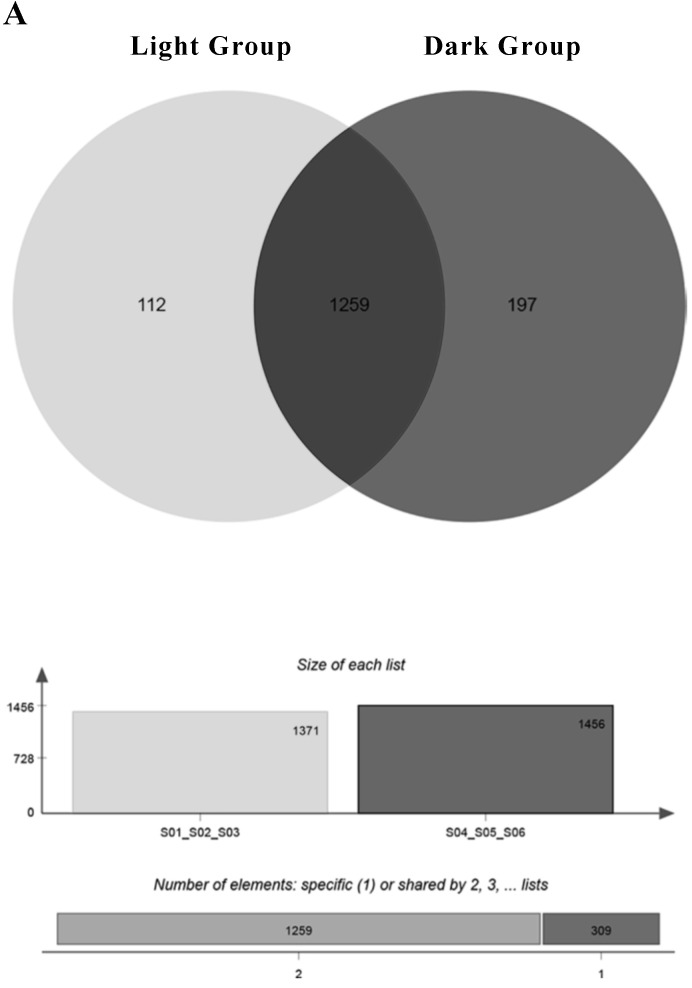
The miRNAs that are expressed in all three replicates of a group (light or dark).

A total of 1989 miRNAs were detected using miRDeep2, including 638 known miRNAs and 1351 newly predicted miRNAs (Table 1). Since miRNAs are highly conserved among species, we classified the detected known miRNAs and new miRNAs into miRNA families based on sequence similarity (Support Table S9). One hundred twelve miRNAs were mainly expressed under light conditions. They were expressed in all light groups and not expressed in at least one dark sample. One hundred ninety-seven miRNAs were mainly expressed under dark conditions, and 1259 miRNAs were expressed in all groups (Figure 3A). The relevant information of miRNA sequencing is explained in Support File 2.

### Enrichment of the differentially expressed circRNAs and miRNAs

**Figure 4:**
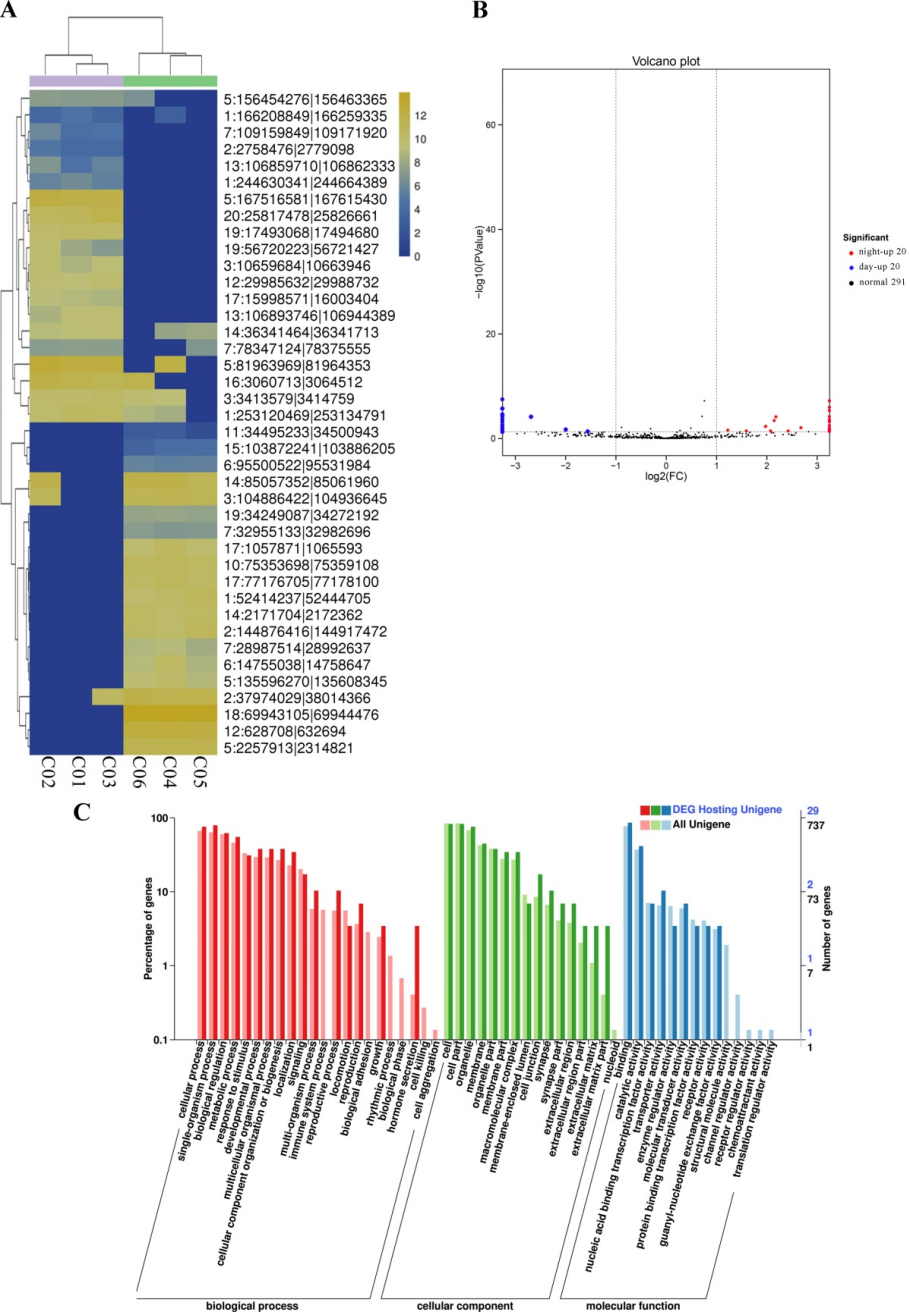
** Differential expression of circRNAs between day and night.** (A) Cluster analysis of the differentially expressed circRNAs. The different columns represent different samples, and the different rows represent different circRNAs. The color represents the level of circRNA expression in the sample (log2TPM+1). (B) Volcano plot of circRNA expression between the two groups. The abscissa represents the logarithm of the difference in circRNA expression between two groups. The ordinate represents the negative logarithm of the P value. The blue dots represent circRNAs with day-up expression, the red dots represent circRNAs with night-up expression, and the black dots represent circRNAs with no significant differences in expression. (C) GO map of all the circRNA host genes and the host genes of the differentially expressed circRNAs. The abscissa shows the GO classification, the left ordinate shows the percentage of genes, and the right ordinate shows the number of genes. All Unigene: the host genes of all circRNAs. DEG Hosting Unigene: the host genes of the differentially expressed circRNAs.

After the expression of all circRNAs and miRNAs was statistically analyzed, the RNA expressed only in individual samples was excluded. Then, DEseq was used to analyze the diurnal differential expression of circRNAs and miRNAs. For comparisons of the processed data between the light and dark groups, a fold change > 2 and *P* value < 0.05 were selected as the criteria for circRNAs with significant differential expression (Figure 4A and Support Table S8). Ultimately, 40 differentially expressed circRNAs were obtained, among which 20 circRNAs were significantly upregulated during the day and 20 circRNAs were significantly upregulated during the night (Figure 4B and Table 2). We performed GO and KEGG enrichment analyses for all the circRNA host genes and for the host genes of the differentially expressed circRNAs. The GO enrichment results are presented in Figure 4C and Support Table S7. In addition, a topGO-directed acyclic graph was constructed with the enriched terms. Ultimately, 29 circRNA host genes were annotated with GO terms, including “protein serine/threonine kinase activity” (GO: 0004674) and “ion channel activity” (GO: 0005216). The results of the KEGG pathway enrichment analysis of the differentially expressed circRNA host genes are shown in Support Table S7. Twenty of the genes were enriched in the KEGG pathways bacterial invasion of epithelial cells, bile secretion and CoA biosynthesis. All data for differentially expressed circRNAs are provided in Support Tables S7-8.

**Figure 5:**
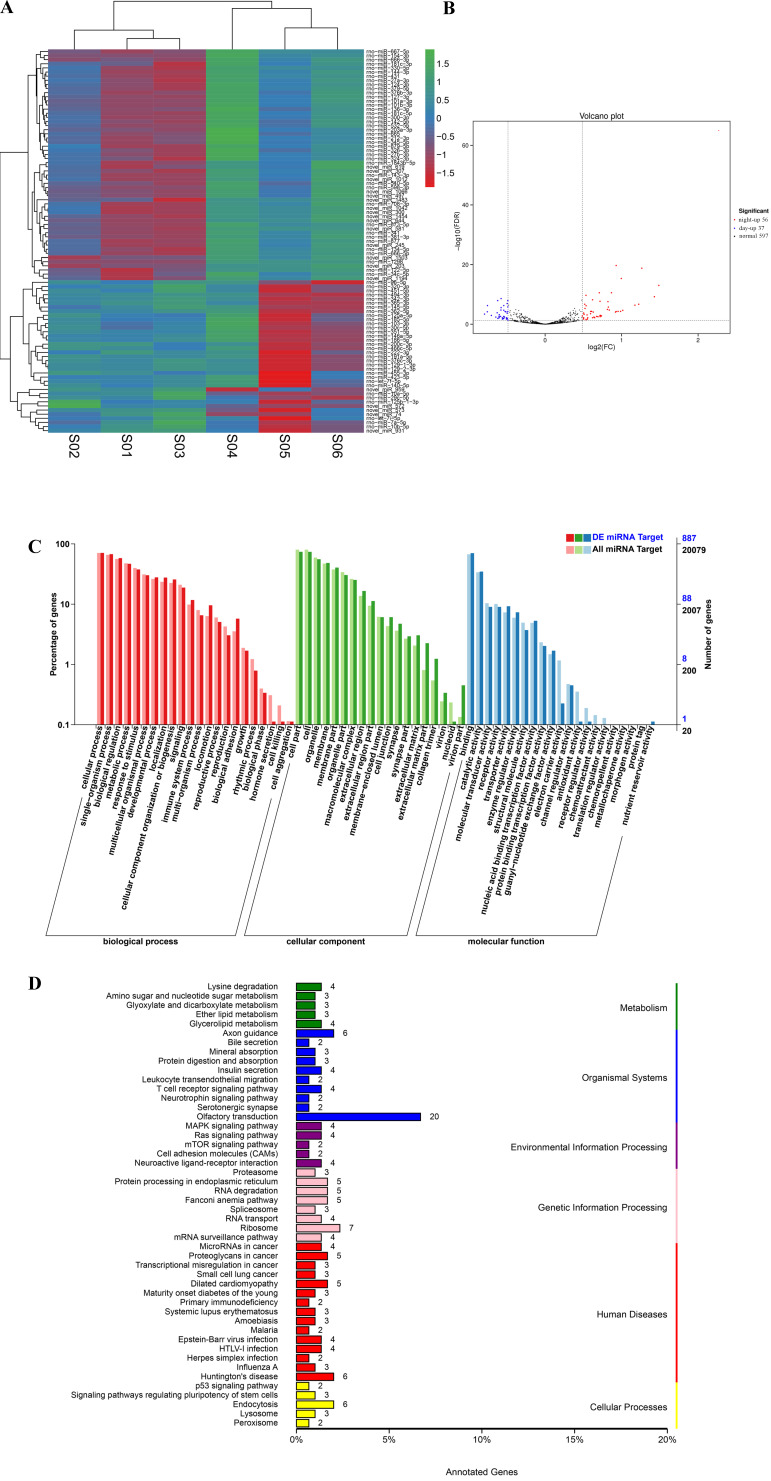
** Differential expression of miRNAs between day and night.** (A) Cluster analysis of the differentially expressed miRNAs. The different columns represent different samples, and the different rows represent different miRNAs. The color represents the level of miRNA expression in the sample (log2TPM+1). (B) Volcano plot of miRNA expression between the two groups. The abscissa represents the logarithm of the difference in miRNA expression between two groups. The ordinate represents the negative logarithm of the *FDR*. The blue dots represent miRNAs with day-up expression, the red dots represent miRNAs with night-up expression, and the black dots represent miRNAs with no significant differences in expression. (C) GO map of all the miRNA target genes and the differentially expressed genes. The abscissa indicates the GO classification, the left ordinate indicates the percentage of genes, and the right ordinate indicates the number of genes. DE miRNA Target: the miRNA targeting genes with differential expression. All miRNA Target: all the miRNA targeting genes. (D) KEGG classification map of the miRNA target genes. The ordinate lists the name of the KEGG metabolic pathway, and the abscissa shows the number of miRNA target genes annotated to the pathway and the proportion among the total number of genes annotated.

Similarly, for comparisons of the processed data between the day and night groups, a fold change > 0.5 and *FDR*<0.01 were selected as the criteria for miRNAs with significant differential expression (Figure 5A and Support Table S10). Ultimately, 93 differentially expressed miRNAs were obtained, among which 37 miRNAs were significantly upregulated during the day and 56 miRNAs were significantly upregulated during the night (Figure 5B and Table 3). We performed GO and KEGG enrichment analyses of all the miRNA target genes and the target mRNAs of the differentially expressed miRNAs. The GO enrichment results are presented in Figure 5C. In addition, a topGO-directed acyclic graph was constructed with the enriched terms. Ultimately, 887 miRNA target genes were annotated with the GO terms “positive regulation of transcription” (GO: 0045944), “G-protein coupled receptor signaling pathway” (GO: 0007186), “G-protein coupled receptor activity” (GO: 0004930) and “nucleolus” (GO: 0005730). The results of the KEGG pathway enrichment analysis of the differentially expressed genes are shown in Figure 5D. Five hundred forty target genes were enriched in the KEGG pathways Jak-STAT signaling pathway, mRNA surveillance pathway, spliceosome and protein processing in the endoplasmic reticulum. All data for differentially expressed miRNAs and the analysis of the miRNAs are provided in Support Tables S10-12.

### Relative expression of circRNAs

**Figure 6:**
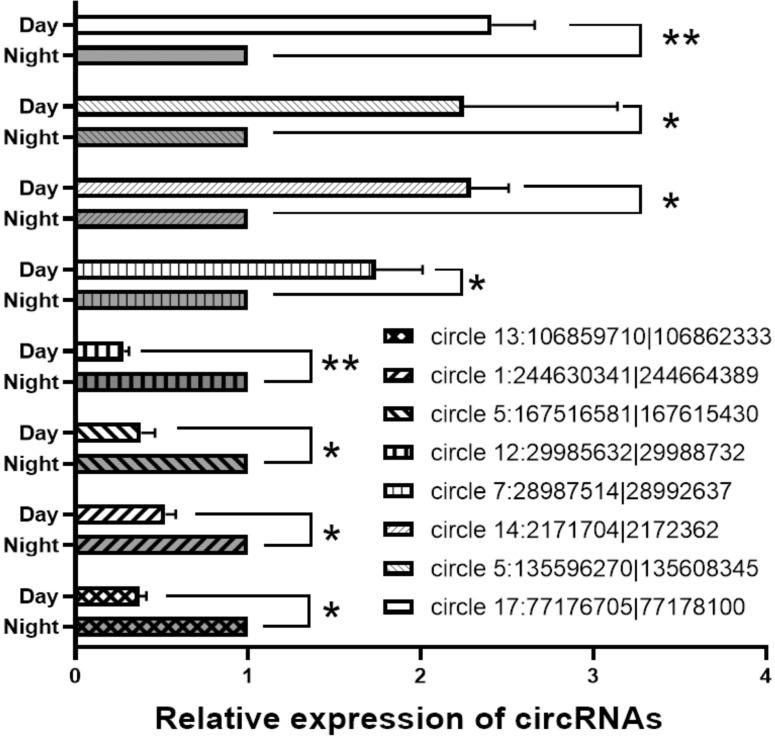
** qPCR results for differentially expressed circRNA.** *, P<0.05. **, P<0.01.

For verification of the circRNA data generated using RNA-seq analysis, we selected 8 circRNAs and then detected their relative expression levels in the pineal gland during the day and night using qPCR. Since the two side sequences of the circRNA splicing site cannot match on the genome, the primers for circRNAs were designed to amplify across circRNA splicing sites with specificity (Support Table S13). As shown in Figure 6, these circRNAs were significantly differentially expressed in the rat pineal gland between day and night.

### Interactions between circRNAs, mRNAs and miRNAs

**Figure 7:**
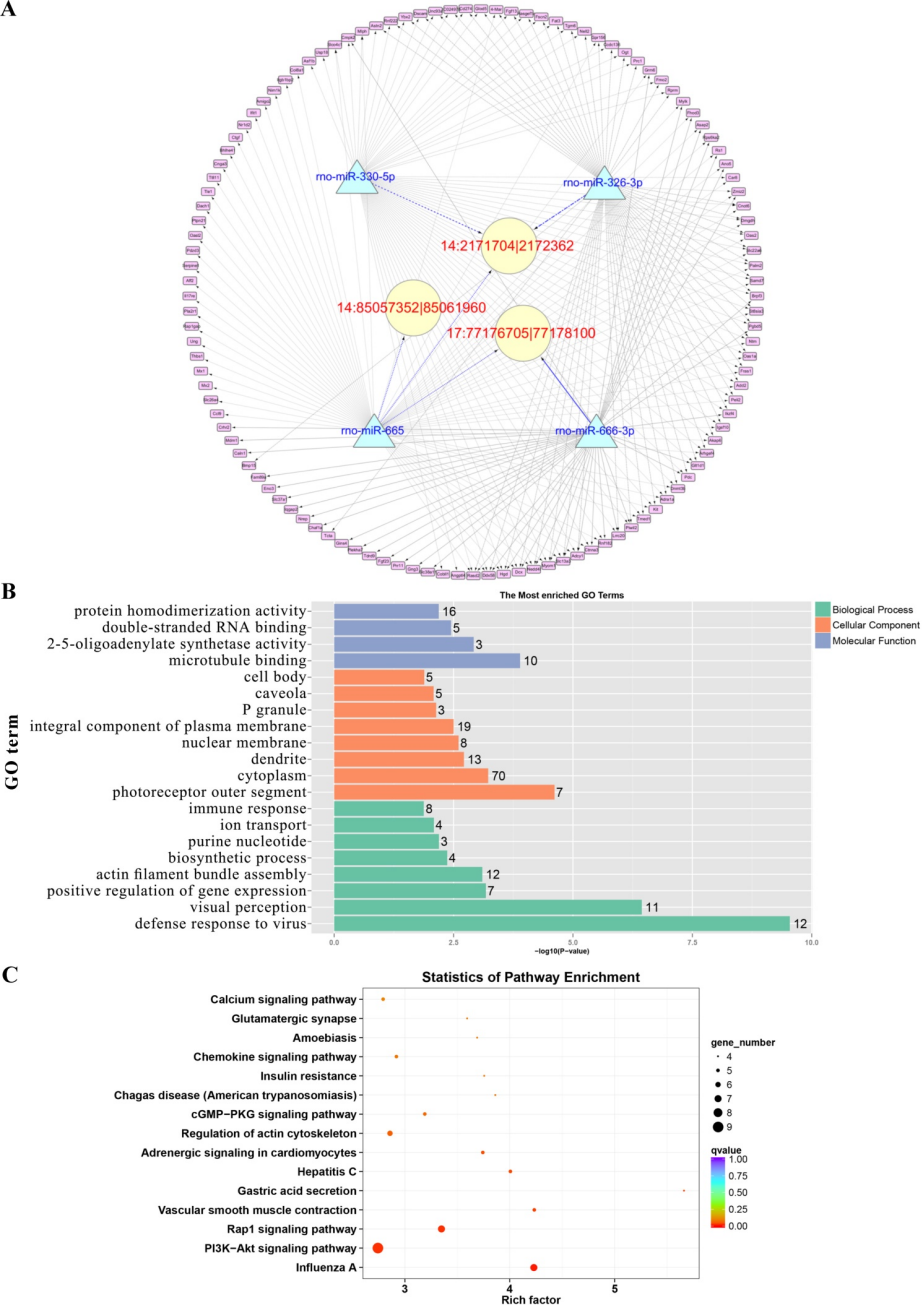

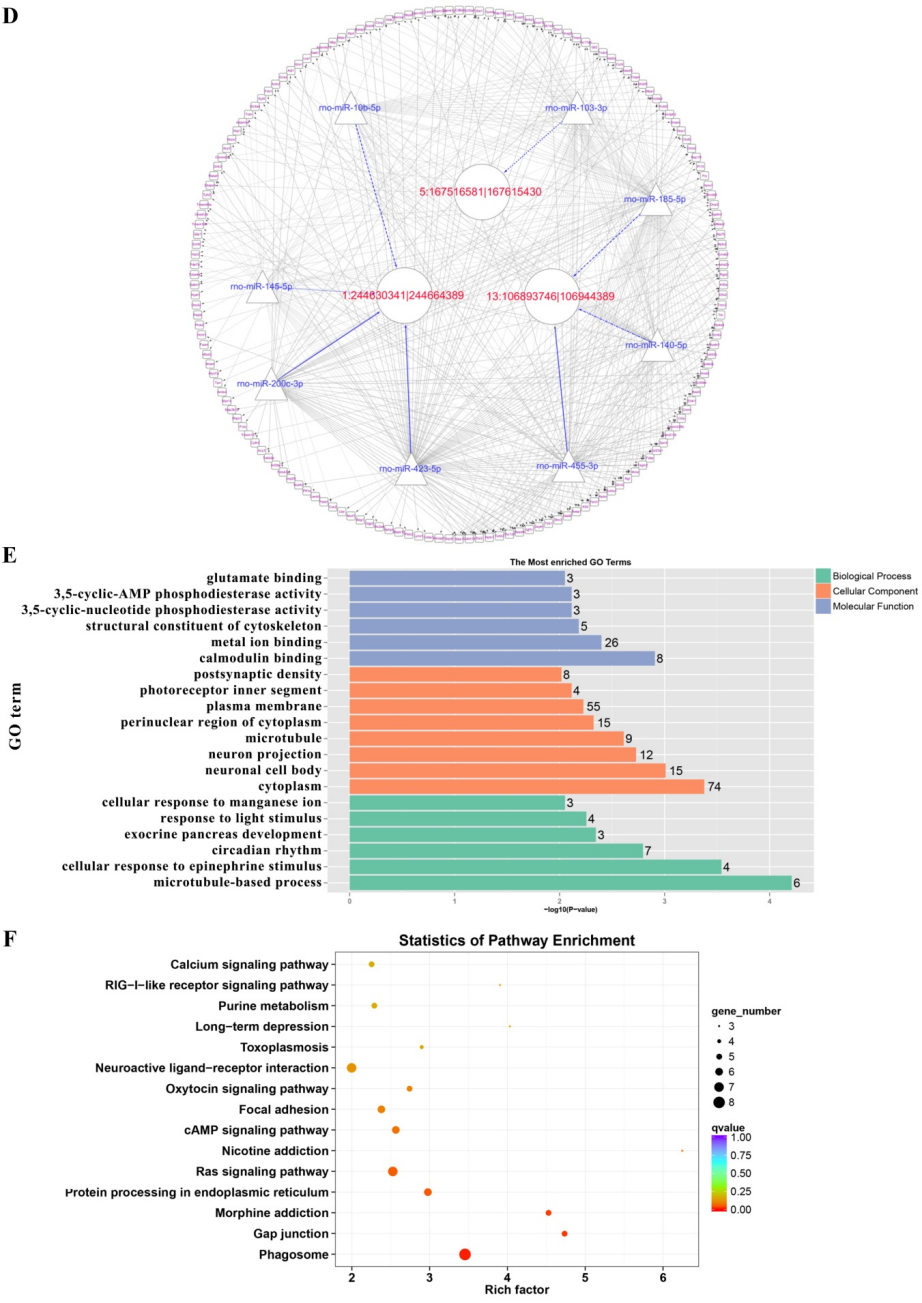
** Prediction of the targeting relationships among miRNAs, mRNAs and circRNAs with differential expression.** (A) The daytime ceRNA network. The triangles represent miRNAs, the circles represent circRNAs, the rectangles represent mRNAs, the blue lines indicate targeting relationships between miRNAs and circRNAs, and the black lines indicate targeting relationships between miRNAs and mRNAs. (B) GO map of all the miRNA target genes in the daytime ceRNA network. (C) KEGG classification map of the miRNA target genes in the daytime ceRNA network. (D) The night-time ceRNA network. (E) GO map of all the miRNA target genes in the night-time ceRNA network. The left ordinate lists the GO classification, the abscissa shows the percentage of genes, and the right ordinate indicates the number of genes. (F) KEGG classification map of the miRNA target genes in the night-time ceRNA network. The ordinate lists the names of the KEGG metabolic pathways, and the abscissa shows the number of miRNA target genes annotated to the pathway and the proportion among the total number of genes annotated.

Since circRNAs and mRNAs contain multiple miRNA binding sites, miRNAs that may target circRNAs and mRNAs were predicted by the software. The circRNAs, miRNAs and mRNAs we used were identified as differentially expressed. Notably, 6222 interactions among 20 night upregulated circRNAs, 37 night downregulated miRNAs and 205 night upregulated mRNAs were predicted as the ceRNA network in the rat pineal gland at night, and 9718 interactions among 20 day upregulated circRNAs, 53 day downregulated miRNAs and 195 day upregulated mRNAs were predicted as the ceRNA network during the day time. The day and night circRNA-miRNA-mRNA regulatory networks were generated using Cytoscape software to further elucidate the underlying mechanism of the differentially expressed circRNAs we identified (Figure 7A and D).

### GO analysis and KEGG pathway analysis were conducted to explore the biological functions of the ceRNA target genes

For the daytime ceRNA network, the GO analysis revealed that the terms with the most genes and the terms with the highest enrichment scores were both “response to virus” (GO: 0009615) in the biological process category. For the cellular component category, the term “cytoplasm” (GO: 0005737) included the most genes, and “photoreceptor outer segment” (GO: 0001750) was the most significantly enriched term. Furthermore, “protein homodimerization activity” (GO: 0042803) was related to the most genes, and the term with the most significant enrichment was “microtubule binding” (GO: 0008017) in the molecular function category (Figure 7B). Moreover, the enriched KEGG pathways were the PI3K-Akt signaling pathway, Rap1 signaling pathway, adrenergic signaling in cardiomyocytes, and cGMP-PKG signaling pathway, among others. These pathways were related to the transmission of light signals and regulation of neuroendocrine signals (Figure 7C).

For the night ceRNA network, the GO analysis revealed that the term with most genes and the term with the highest enrichment score were both “cytoplasm” (GO: 0005737). For the biological process and molecular function categories, the terms “cellular response to epinephrine stimulus” (GO: 0071872), “circadian rhythm” (GO: 0007623), “response to light stimulus” (GO: 0009416), and “3',5'-cyclic-AMP phosphodiesterase activity” (GO: 0004115) were related to melatonin secretion and circadian rhythm in the pineal gland (Figure 7E). Moreover, the enriched KEGG pathways were phagosome, gap junction, morphine addiction, protein processing in endoplasmic reticulum, Ras signaling pathway, and cAMP signaling pathway, among others (Figure 7F).

## Discussion

The pineal gland is the main endocrine organ that secretes melatonin, a biological circadian signal that plays an important regulatory role in animal fitness 39. Changes in noncoding RNAs in the pineal gland are considered an important component of the circadian rhythm mechanism 40. According to previous studies, lncRNAs exhibit differential expression in the pineal gland between day and night and are believed to be involved in circadian biology 41. miR-483 and miR-325-3p were also found to be involved in circadian rhythm regulation by inhibiting AANAT expression 13, 14. At the same time, as a member of the ncRNA family, circRNAs have been increasingly shown to play important roles in various cellular functions 42. However, studies of circRNAs in the rat pineal gland have rarely been reported. Only one study reported significant differences in circRNA expression in the pineal gland of AD mice 43. Therefore, in our study, RNA-seq was used to identify and detect the expression of mRNAs, circRNAs and miRNAs in the rat pineal gland between day and night. Finally, we identified 40 circRNAs and 97 miRNAs that were differentially expressed. And at the same time, the lengths and distributions of all circRNAs on chromosomes were sort out, which may provide the data basis for further targeted studies of circRNAs.

For the miRNAs in the pineal gland, previous study showed that miR-182, miR-183, miR-127 and miR-127 was the 4 most abundant pineal miRNAs. And pinealocytes transfected with these miRNAs showed no effect on melatonin synthesis. In our research, miR-182 and miR-183-5p are also the most abundant miRNAs. For more and more miRNAs are annotated in the miRBase, other miRNAs are found with a high level of expression in our research. Such as the let-7 fimally, which is mainly expressed in mammalian brains, more members of it are found in the pineal gland than before. And some of them are significantly changed between day and night. As miRNAs are involved in transcriptional regulation of mRNA, the potential functions of miRNAs can be interpreted by predicting the target genes. According the predicted results, we can purposefully transfect miRNAs and verify their physiological functions.

Typically, circRNAs function as miRNA sponges to competitively adsorb microRNAs and regulate intracellular gene expression, which is known as the ceRNA mechanism 44. We examined circRNA-miRNA and miRNA-mRNA interactions and discovered that each selected circRNA that contained at least one miRNA binding site was able to interact with several miRNAs. Then, the differentially expressed circRNAs, miRNAs and mRNAs were selected to draw a complete picture of ceRNA regulatory networks. This circRNA-miRNA-mRNA network may provide insights into the regulatory pathways in the pineal gland or the cascade-amplifying synergistic effects of circRNA-miRNA and miRNA-mRNA networks. This network provided theoretical support that circRNAs probably played a role in circadian rhythms by indirectly targeting certain mRNAs.

Furthermore, a GO enrichment analysis and KEGG pathway analysis were also performed to functionally annotate the predicted target genes. For the daytime ceRNA network, GO analysis revealed the enrichment of the terms “photoreceptor outer segment” and “protein homodimerization activity”, which are related to adrenergic receptors 45 and rhythmic pineal protein/DNA binding transcription factors 46. The KEGG pathway analysis revealed several important pathways related to the circadian clock, including the PI3K-Akt signaling pathway, adrenergic signaling in cardiomyocytes and cGMP-PKG signaling pathway. The PI3K-Akt signaling pathway has been shown to be regulated by the clock proteins Cry1, Cry2 and Bmal1, which are part of the oscillations of the transcriptional-translational negative feedback system and regulate the circadian rhythm 43, 47, 48. Mustafa C Beker reported an interaction of melatonin and Bmal1 in the regulation of PI3K/AKT pathway components and cellular survival 49. Moreover, the inhibition of cGMP phosphodiesterase enhances photic responses and the synchronization of the biological circadian clock in rodents 50. For the night-time ceRNA network, the GO analysis revealed enrichment of the terms “cellular response to epinephrine stimulus”, “circadian rhythm” and “3',5'-cyclic-AMP phosphodiesterase activity”, which are related to melatonin secretion. KEGG pathway analysis revealed several important pathways related to melatonin secretion, including the cAMP signaling pathway 51, 52. Altogether, GO and KEGG analyses suggested that circRNAs with different expression may participate in the process of pineal biological function via different pathways.

In summary, this is the first study to profile the transcriptome of the rat pineal gland from day and night. We constructed a ceRNA network of circRNAs, miRNAs, and mRNAs, which may play a key regulatory role in the circadian rhythm and melatonin secretion of the pineal gland. We expect that our study will be helpful for researchers interested in the gene expression change of the pineal gland with circadian rhythm. Although we identified differentially expressed RNAs between day and night, more experiments are needed at different time points to prove circadian changes. In the future, additional deep-seated regulatory mechanisms await discovery, and further research on them may expose potential therapeutic targets for circadian rhythm diseases. Ideally, our future studies will construct circRNA regulation pathways.

## Supplementary Material

Supplementary figures and table S1.

Supplementary tables.

## Figures and Tables

**Table 1:** The number of miRNAs expressed in the sample

Sample-ID	Known-miRNAs	Novel-miRNAs	Total
S01	568	1065	1633
S02	579	1128	1707
S03	565	1058	1623
S04	586	1161	1747
S05	572	1072	1644
S06	579	1116	1695
**Total**	**638**	**1351**	**1989**

**Table 2:** The top 10 circRNAs with the most significant differentially expressed

#ID	circRNA length	P-Value	log2FC	regulated
17:77176705|77178100	380	0.000	-Inf	day-up
6:95500522|95531984	31463	0.000	-2.176	day-up
1:52414237|52444705	329	0.000	-Inf	day-up
19:34249087|34272192	23106	0.000	-Inf	day-up
12:628708|632694	313	0.000	-2.142	day-up
7:32955133|32982696	27564	0.002	-Inf	day-up
7:28987514|28992637	568	0.004	-Inf	day-up
14:2171704|2172362	359	0.004	-Inf	day-up
17:1057871|1065593	7723	0.005	-Inf	day-up
3:104886422|104936645	50224	0.006	-1.951	day-up
1:244630341|244664389	1749	0.000	Inf	night-up
13:106859710|106862333	327	0.000	Inf	night-up
13:106893746|106944389	1800	0.000	Inf	night-up
5:167516581|167615430	762	0.000	Inf	night-up
20:25817478|25826661	9184	0.000	Inf	night-up
17:15998571|16003404	673	0.000	Inf	night-up
16:3060713|3064512	566	0.000	2.687	night-up
3:10659684|10663946	399	0.001	Inf	night-up
12:29985632|29988732	353	0.001	Inf	night-up
7:109159849|109171920	12072	0.002	Inf	night-up

**Table 3:** The top 10 miRNAs with the most significant differentially expressed

#ID	Pvalue	FDR	log2FC	regulated
novel_miR_581	0.000	0.000	2.270	night-up
rno-miR-341	0.000	0.000	0.926	night-up
rno-miR-1298	0.000	0.000	1.266	night-up
rno-miR-154-3p	0.000	0.000	0.996	night-up
rno-miR-122-5p	0.000	0.000	1.485	night-up
rno-miR-124-3p	0.000	0.000	0.870	night-up
rno-miR-431	0.000	0.000	0.706	night-up
rno-miR-101a-3p	0.000	0.000	0.535	night-up
novel_miR_644	0.000	0.000	1.428	night-up
rno-miR-666-5p	0.000	0.000	0.621	night-up
rno-miR-376c-3p	0.000	0.000	-0.578	day-up
rno-miR-342-3p	0.000	0.000	-0.621	day-up
rno-miR-191a-5p	0.000	0.000	-0.486	day-up
rno-miR-129-1-3p	0.000	0.000	-0.494	day-up
rno-miR-129-2-3p	0.000	0.000	-0.494	day-up
rno-miR-125b-1-3p	0.000	0.000	-0.476	day-up
rno-miR-365-3p	0.000	0.000	-0.755	day-up
rno-miR-494-3p	0.000	0.000	-0.543	day-up
rno-miR-451-5p	0.000	0.000	-0.491	day-up
rno-miR-10b-5p	0.000	0.000	-0.577	day-up

## Abbreviations

MT: melatonin
cAMP: cyclic adenosine monophosphate
NE: norepinephrine
AANAT: arylalkylamine N-acetyltransferase
ASMT: acetylserotonin O-methyltransferase
ceRNA: competitive endogenous RNA
CIRI: CircRNA identifier
TPM: transcripts per kilobase million
FDR: false discovery rate
GO: Gene Ontology
KEGG: Kyoto Encyclopedia of Genes and Genomes
