# Supplementary material for: Identifying daily changes in circRNAs and circRNA-associated-ceRNA networks in the rat pineal gland
Source: Int J Med Sci. 2021 Jan 14;18(5):1225–39. doi: 10.7150/ijms.51743 (PMC7847611; doi:10.7150/ijms.51743)
Supplement: Supplementary file 1 — Supplementary figures and table S1. [file ijmsv18p1225s1.pdf]

Support File 1：The differentially expressed mRNAs between day and night

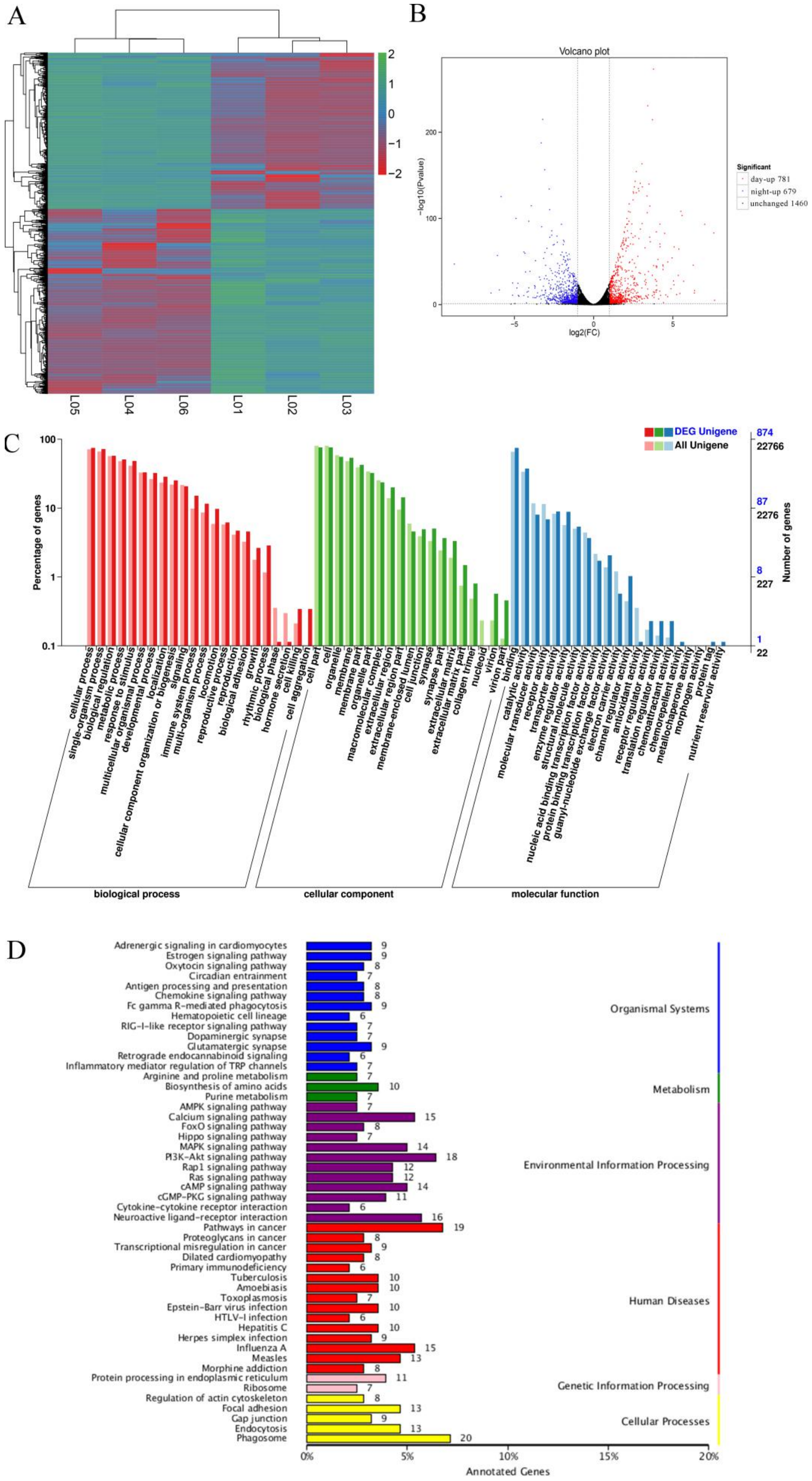

(Figure 1. Differential expression of mRNA during circadian cycles. (A) Cluster analysis of the differentially expressed mRNAs. The different columns represent different samples, and the different rows represent different mRNAs. The color represents the level of mRNA expression in the sample (log2FPKM+1). (B) Volcano plot of mRNA expression between the two groups. The abscissa represents the logarithm of the difference in mRNA expression between two groups. The ordinate represents the negative logarithm of the P value. The blue dots represent mRNAs with night-up expression, the red dots represent mRNAs with day-up expression, and the black dots represent mRNAs with no significant differences in expression. (C) GO map of all the miRNA target genes and the differentially expressed genes. The abscissa indicates the GO classification, the left ordinate indicates the percentage of genes, and the right ordinate indicates the number of genes. (D) KEGG classification map of the genes. The ordinate lists the name of the KEGG metabolic pathway, and the abscissa shows the proportion among the total number of genes annotated.**)**

Table 1: The top 10 mRNAs with the most significant differentially expressed

#ID	gene_symbol	FDR	log2FC	regulated
ENSRNOG00000017659	Hs3st2	2.99E-112	3.936848229	day-up
ENSRNOG00000008020	Pax4	1.12E-111	3.309473501	day-up
ENSRNOG00000056772	Col4a6	2.12E-95	3.085314078	day-up
ENSRNOG00000006084	Cngb3	2.75E-81	2.798990826	day-up
Rat_newGene_208107	Rat_newGene_208107	8.10E-76	Inf	day-up
Rat_newGene_21747	Rat_newGene_21747	5.22E-67	5.838430288	day-up
Rat_newGene_214530	Rat_newGene_214530	5.31E-67	Inf	day-up
Rat_newGene_57294	Rat_newGene_57294	7.66E-65	2.776258016	day-up
Rat_newGene_95991	Rat_newGene_95991	5.07E-58	3.080651906	day-up
ENSRNOG00000018185	Sag	3.71E-54	3.216221995	day-up
Rat_newGene_191842	Rat_newGene_191842	0	-6.906956331	night-up
ENSRNOG00000033502	Ribc2	6.05E-283	-6.33803842	night-up
ENSRNOG00000009177	Fcer1a	4.05E-228	-5.277819233	night-up
ENSRNOG00000011598	Slc15a1	6.98E-216	-6.296901293	night-up
ENSRNOG00000018268	Hhip	2.28E-209	-5.111714671	night-up
ENSRNOG00000012878	Atp7b	1.11E-182	-6.601563623	night-up
ENSRNOG00000008911	Draxin	5.13E-156	-4.449908943	night-up
ENSRNOG00000033026	Dclk3	3.52E-131	-3.714942946	night-up
ENSRNOG00000011182	Aanat	4.17E-122	-6.158042258	night-up
ENSRNOG00000021817	Irak2	7.75E-97	-3.051272716	night-up

DEseq was used to analyze the diurnal differential expression of mRNAs. For comparisons of the processed data between the light and dark groups, a fold change > 2 and FDR< 0.05 were selected as the criteria for mRNAs with significant differential expression (Figure 1A and Support Table 14). Ultimately, 1460 differentially expressed mRNAs were obtained, among which 781 mRNAs were significantly upregulated during the day and 679 mRNAs were significantly upregulated during the night (Figure 1B and Table 1). We performed GO and KEGG enrichment analyses for all the mRNA and for the differently expressed mRNAs. The GO enrichment results are presented in Figure 1C and Support Table 17. Ultimately, 874 genes were annotated with GO terms, including “catalytic activity” and “cellular process”. The

results of the KEGG pathway enrichment analysis of the differentially expressed mRNAs are shown in Support Table 18. Two hundred and eighty of the genes were enriched in the KEGG pathways cAMP signaling pathway, PI3K-Akt signaling pathway and Neuroactive ligand-receptor interaction. All data for differentially expressed mRNAs is provided in Support Tables 17-18.

Support File 2：

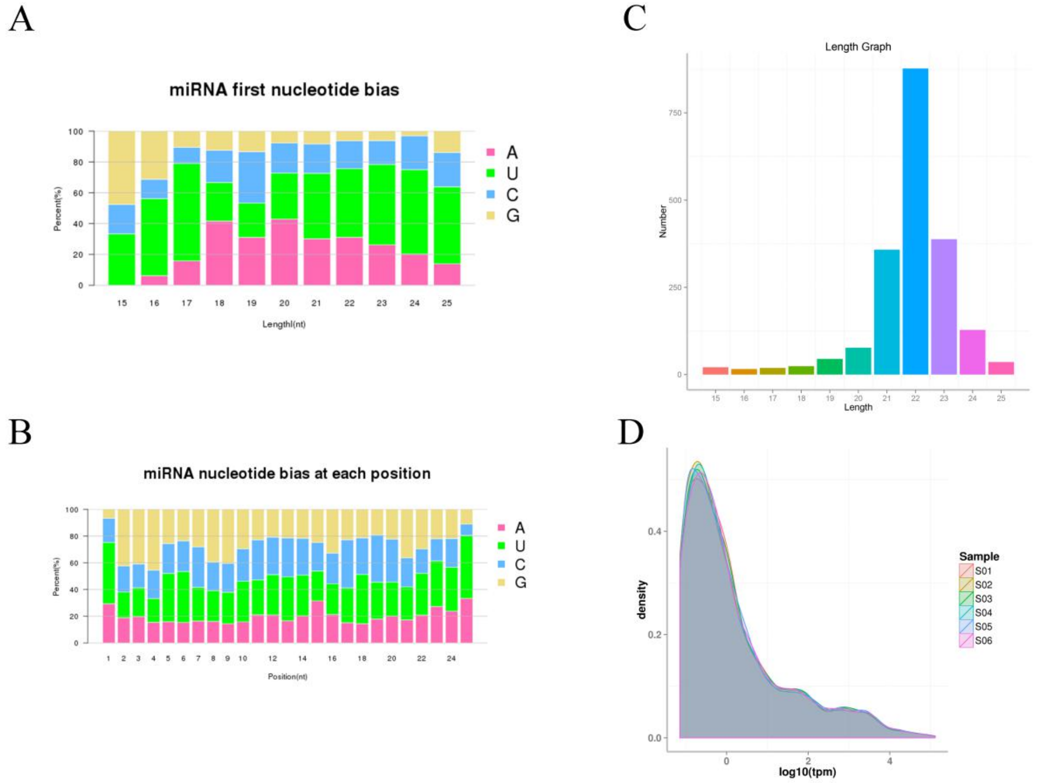

(**Figure 2. miRNA expression in the pineal gland.** (A) First-nucleotide bias of all miRNAs in the pineal gland. (B) Nucleotide bias at each position for all miRNAs. (C) Lengths of all miRNAs. (D) Distribution of the expression of all miRNAs.)

Since miRNAs are highly conserved among species, we classified the detected known miRNAs and new miRNAs into miRNA families based on sequence similarity. The base preference at each position in the whole miRNA was statistically analyzed, and U accounted for most of the 5'-terminal bases, in line with the typical proportions in miRNAs (Figure 3.A.B). The final lengths of most of the mature miRNAs were in the range of 20 nt to 24 nt, and the average overall length of the identified miRNAs was 22 nt (Figure 3.C.D).
